# Spatial confinement governs orientational order in patchy particles

**DOI:** 10.1038/srep27599

**Published:** 2016-06-06

**Authors:** Yasutaka Iwashita, Yasuyuki Kimura

**Affiliations:** 1 Department of Physics, Kyushu University, 819-0395 Fukuoka, Japan

## Abstract

Orientational order in condensed matter plays a key role in determining material properties such as ferromagnetism, viscoelasticity or birefringence. We studied purely orientational ordering in closely-packed one-patch colloidal particles confined between flat substrates, where the particles can only rotate and are ordered *via* the sticky interaction between the patches. For the first time, we experimentally realized a rich variety of mesoscopic patterns through orientational ordering of colloids by controlling patch size and confinement thickness. The combination of experiment and numerical simulation reveals the decisive role of confinement: An ordered state(s) is selected from the (meta)stable options in bulk when it is commensurate with the system geometry and boundary conditions; otherwise, frustration induces a unique order. Our study offers a new means of systematic control over mesoscopic structures *via* orientational ordering in patchy particles. The system would also possess unique functionalities through the rotational response of the particles to external stimuli.

Material properties of condensed matter owe a great part to the *positional* and *orientational* order of unit structures^1^. For example, crystalline phases are defined by positional order, usually accompanied by orientational order of the units in e.g. atomic/molecular crystals. On the other hand, in the simplest cases, magnetic or dielectric phases are characterised by purely orientational ordering of the dipoles. In liquid crystal phases, the variety of (de)couplings between those order parameters gives rise to unique optical, rheological properties, etc. The diverse combinations of orders in condensed matter predominantly originate from the anisotropy in interaction between the units it is made from, such as directional bonding between atoms/molecules, dipolar interaction, or steric interaction derived from shape anisotropy of molecules.

At the mesoscale, the fundamental roles of anisotropy in unit structures i.e. colloidal particles have recently attracted significant interest in the research community^2^,^3^: For designing self-assembled mesostructures with novel photonic, plasmonic, catalytic functions etc., diverse anisotropic nanoparticles have been synthesised as building blocks. For proteins, as typical biological colloids, the anisotropic shape and surface chemistry play key roles in their collective behaviour^4^,^5^,^6^.

Recently, patchy colloidal particles have undergone rigorous study as an ideal model system with a well-defined interaction anisotropy^7^. These particles possess sticky patches on their surfaces (Fig. 1), their positions and size dictating the directions and number of inter-patch attractive bonds. Thus, they have been called *molecular colloids*, and interesting results specifically due to their anisotropy have been reported; e.g. new colloidal crystal phases such as a kagome lattice^8^,^9^, equilibrium gel phases^10^,^11^, and phase behaviour closely related to protein solutions^5^, making them a powerful mesoscopic model system. However, the primary interest of most of these undertakings has been in positional order or phase behavior in density space, despite the fundamental significance of orientational order described above.

A colloidal system exhibiting purely orientational ordering recently has been studied in closely-packed spherical patchy particles^12^,^13^,^14^,^15^,^16^, where the particle motion is almost purely rotational (Fig. 1) and the particles are ordered by the inter-patch attraction. For the bulk system, numerical works have reported a rich phase behaviour by the order^12^,^13^. The experimental realization has been only for a hexagonal monolayer^15^,^16^, whose phase behaviour agrees qualitatively with the corresponding simulation^14^,^16^. Orientational order is expected to render unique functionalities to the mesosystem compared with positional order in a conventional colloidal system as the case in atomic/molecular condensed matter describe above, in addition to offering a new means of systematic control over mesoscopic patterns with various types of symmetry and dimensionalities. The phase behavior is, however, inherently complex: A patch size often gives rise to multiple states with identical internal energy, i.e. degeneracy in ground states, resulting in multiple (meta)stable states with similar free energy^12^,^13^,^14^,^16^.

In this contribution, therefore, we study the purely orientational ordering in multiple layers of closely-packed spherical one-patch particles confined between flat substrates both in experiment and numerical simulation. The results show a great role of spatial confinement in the ordering: A state which is commensurate with the geometry and boundary conditions of confinement is selected from the degenerate options in a bulk system, or even a unique state appears when none of those options in bulk is commensurate.

In our experiment (see Materials and Methods for the details), particles possess an isotropic (circular) gold patch with a half opening angle *θ*_ap _< *π*/2 (Fig. 1). The bonding between the patches is due to the large van der Waals attraction between gold layers^17^. The bonding energy ε, in units of thermal energy *k*_B_*T*, is moderate, which was estimated as ~4 for the experimental system here^16^,^18^. The particles were closely packed into several spatial arrangements induced by the confinement thickness, i.e. layering transitions in a wedge-shaped cell^19^,^20^,^21^; here we focus on two to four layers of tetragonally (□) and hexagonally (Δ) packed particles, appearing sequentially as 2□ → 2Δ → 3□ → 3Δ → 4□ → 4Δ with increasing the ratio of sample thickness to particle diameter, *h*/*d*. Due to the packing, the particle motion is almost purely rotational (Fig. 1), and there is no noticeable preference of particle orientation with respect to the substrate. We observed the steady structures after the relaxation following the packing: A structure fluctuates thermally with active recombination of inter-patch bonds because of the moderate ε. The early process of the ordering occurs on the packing under centrifugation and depends on the packing kinetics (see Materials and Methods), thus not considered here.

We also performed a Monte Carlo simulation with ~1000 particles per monolayer (see Materials and Methods). The simulation assumes purely rotational motion of particles and a simple interaction, i.e. only an inter-patch attraction, which is constant over the area of a patch. Thus, in Fig. 1, an attraction acts only between particles 1 and 2, i.e. they are bonded, while there is no interaction for the other pairs. The maximum possible number of bonds on a patch, 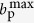, depends solely on *θ*_ap_ ; the larger the patch, the more bonds that can be formed^14^ (Supplementary Note 2). The results with this simple model are compared with the experiment for discussing the essential mechanism of the ordering.

## Results

### Characteristic patterns by orientational ordering

**Figure 2:**
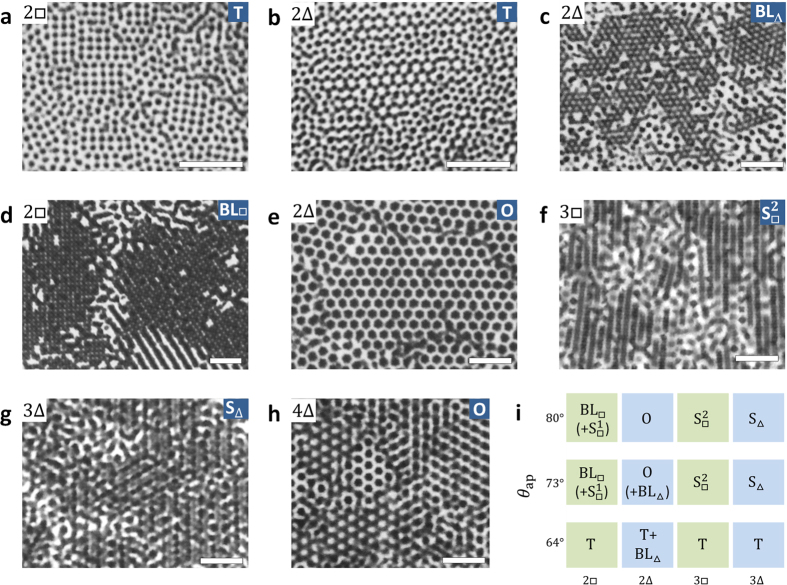
Characteristic patterns dependent on sample thickness and patch size. (**a**–**h**) Optical microscopy images, with particle arrangements given in the upper left, predominant orientationally-ordered states in the upper right; the structural details are described in the following sections. The minor regions of isolated domains in (**c**) correspond to T state. The striped pattern around the bottom-right part in (**d**) corresponds to 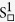 state. A patch appears dark, and particle shape is not resolved in optical microscopy images. For visual comparison, the apparent size of a particle is equalized each other in the images by scaling them. (**i**) The predominant states observed in the experiment for the particle diameter *d* = 1.5 μm. An ordered structure whose fraction is minor is shown in parentheses. “+” denotes the coexistence of a structure. For *θ*_ap_ = 73° and 80°, the predominance of a pattern is higher for 80°. *d* = 1.0 μm and *θ*_ap_ = 70° for (**a**,**b**), and *d* = 1.5 μm and *θ*_ap_ = 64° for (**c**,**f**), *θ*_ap_ = 73° for (**e**,**g**,**h**), *d* = 2.0 μm and *θ*_ap_ = 76° for (**d**). Scale bars are 10.0 μm.

Firstly, we show characteristic patterns observed with optical microscopy (Fig. 2): Their structural details and the formation mechanisms are described in the following sections. Apparently, there is a great variety of patterns of dark domains, i.e. gold patches; zero-dimensional (isolated) domains with four, three and six-fold local rotational symmetry in the domain alignment in Fig. 2a,b,e respectively, a striped pattern of one-dimensional (linear) domains in Fig. 2f,g, and two-dimensionally (planarly) spanned domains with six and four-fold rotational symmetry in them in Fig. 2c,d respectively. For Fig. 2h, the heterogeneity in pattern reflects the stacking order of hexagonal layers, described later. The thermal fluctuation in a pattern was directly observed under the microscopy, reflecting moderate ε described above. The fluctuation is also a cause of the disturbance in pattern, together with the slight amount of flawed particles (see Materials and Methods).

Figure 2i summarizes predominant patterns which appeared in more than half of the observed area at each condition for *d* = 1.5 μm, where the results for four layers are omitted because the states are almost identical to those for bilayer. This diagram clearly shows the dependence of patterns on patch size and particle arrangement determined by *h*/*d*. For the small patch size, *θ*_ap_ = 64°, most of the patterns are made of isolated small domains (T state), whereas for the large two patch sizes the morphology of domains and their alignment are strongly dependent on particle arrangement.

### Orientationally-ordered states for a small patch

**Figure 3:**
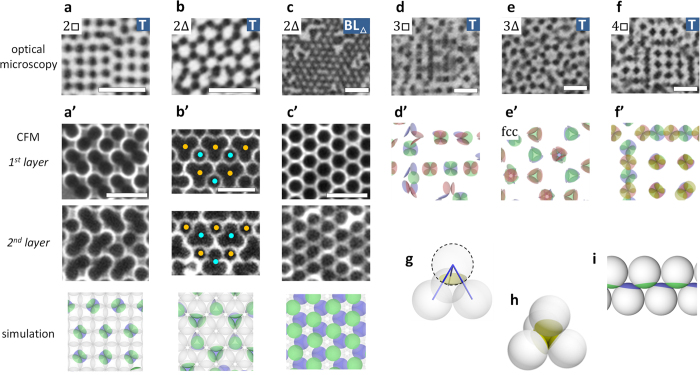
Typical orientationally-ordered structures observed for particles with a small patch, corresponding to 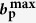 = 3. (**a**–**f**) Optical microscopy images, with particle arrangements and orientationally-ordered states in the corners: T; tetrahedral tetramer, BL; bilayer sheet. (**a**’–**f**’) Magnified images corresponding to (**a–f**), respectively. (**a**’–**c**’) (Top and middle) Laser-scanning confocal microscopy (CFM) images of the first and second layer from the bottom of a sample, respectively. The blue and orange dots in (**b**’) denote the centres of tetramers. A patch is opaque, and a silica surface appears bright with CFM. (Bottom) The corresponding images from simulation showing structural details. (**d**’–**f**’) Simulation images. In those simulation images, all layers are drawn by tuning the transparency of patches and particles for visibility. Patch colours, blue, green, red (pink), and yellow, denote the first, second, third, and fourth layer from the bottom, respectively. (**g**) A schematic drawing of three bonds formed by a patch. (**h**) A tetrahedral tetramer. (**i**) The side view of the simulation image in (**c**’). *d* = 1.0 μm and *θ*_ap_ = 70° for (**a**,**b**), and *d* = 1.5 μm and *θ*_ap_ = 64° for (**c**,**d**,**e**,**f**). *d* = 2.0 μm and *θ*_ap_ = 59° for (**a**’,**b**’,**c**’). Scale bars are 5.0 μm.

Here we describe the states for particles with small patches, such as Fig. 2a–c. Figure 3a–f summarise the change in pattern with the sequential change of particle arrangement. Except for Fig. 3c, patches are bonded into isolated dark domains. The magnified images in Fig. 3a’–f’ show how the patches face each other in the observed patterns. The laser-scanning confocal microscopy (CFM in following) images in Fig. 3a’–c’ show the alignment of opaque patches in each particle layer from the bottom of a cell. In the simulation images in Fig. 3a’–f’, where different patch colours are given to the respective layers, the correspondence of their structures to the optical microscopy and CFM images is observed. In Fig. 3a’ for 2□ and Fig. 3b’ for 2Δ, the cluster formed by inter-patch bonding is a tetrahedral tetramer (Fig. 3h): Two particles of each tetramer belong to a layer in 2□ in Fig. 3a’, and three particles of each tetramer belong to a layer in 2Δ in Fig. 3b’. In a tetramer, all patches can establish three bonds (Fig. 3g–h). Thus, structures filled with tetramers (T state) correspond to ground states for 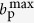 = 3, minimising internal energy.

The translational order in pattern in Fig. 3a,b is local as also seen in Fig. 2a,b, because the tetramers can be packed in different ways without defects: For 2□, the positions of the linearly-aligned tetramers can be shifted by a particle, as seen in Fig. 3a’. For 2Δ, there is another way of packing in addition to the honeycomb structure seen in Fig. 3b,b’ (Supplementary Fig. 3a). With four layers, the order is almost identical to that in two layers: Four layers are made up from the stacking of independent bilayers, as Fig. 3f,f’ for 4□ show. The height difference between patches can be seen from their brightness in the microscopy image and are indicated by their colour in the simulation image. In Fig. 3f’, the tetramers in the upper and lower bilayers overlap at the right-bottom part and not at the other part. For three layers, as in Fig. 3d,e, filling patterns of the tetramers appear less ordered than those in even number of layers; this feature is referred to in the Discussion section.

In addition to the T state, a bilayer sheet structure (BL_Δ_) was found to coexist in 2Δ (Figs 2c and 3c,c’,i). In the structure, all patches can apparently establish three bonds, thus also corresponding to a ground state for 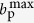 = 3. As described heretofore, orientational order for the small patch is rather simple, consisting solely of tetrahedral tetramers whose space-filling simply reflects particle arrangement, except for the bilayer sheet structure in 2Δ.

### Orientationally-ordered states for a large patch

**Figure 4:**
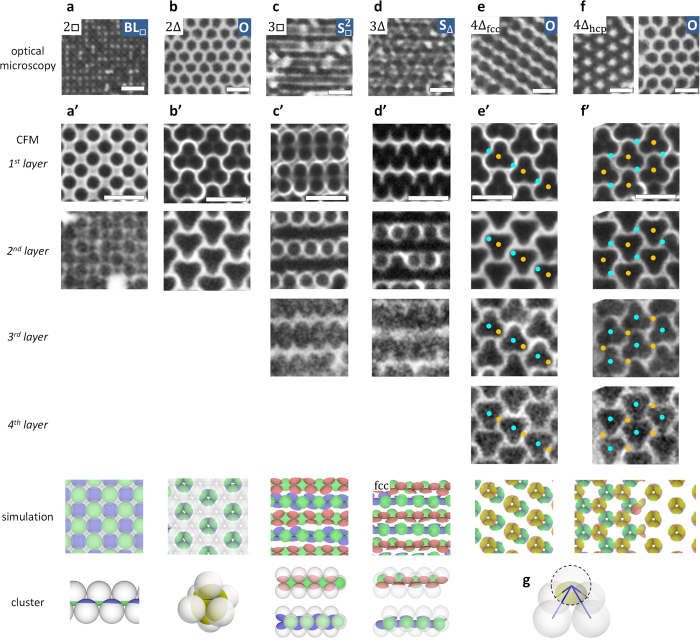
Typical orientationally-ordered structures observed for particles with a large patch, corresponding to 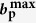 = 4. (**a**–**f**) Optical microscopy images. Particle arrangements and orientationally-ordered states are indicated in the corners: O; octahedral hexamers, S; striped structure. (**a**’–**f**’) Images corresponding to (**a**–**f**), respectively. (Top to bottom) Magnified images of each layer from the bottom of a sample observed with CFM, the corresponding images and cluster structures from simulation, respectively. The orange and blue dots in (**e**’,**f**’) denote the centres of octahedral hexamers in the lower and upper bilayers, respectively, showing the correspondence to the simulation images. For (**f**’), CFM images correspond to the pattern at the left-hand side of the microscopy and simulation images. Note that, with CFM, the image quality of the upper layers is worse, due to the opacity of patches and refraction at particle/fluid interfaces. (**g**) A schematic drawing of four bonds by a patch. *d* = 2.0 μm and *θ*_ap_ = 71°for (**a**’–**c**’,**e**’,**f**’), 76° for (**a**,**d**’). *d* = 1.5 μm and *θ*_ap_ = 73° for (**c**,**e**,**f**), 80° for (**b**,**d**). Scale bars are 5.0 μm.

When a patch is large enough to establish four bonds, i.e. attractively interact with the neighbouring four particles, orientational order provides a much richer variety of phases, as shown in Fig. 4 (see also Fig. 2d–h). In 2□ in Fig. 4a,a’, a bilayer sheet structure (BL_□_) appeared. In 2Δ in Fig. 4b,b’, isolated clusters, octahedral hexamers (O), formed a well-ordered hexagonal lattice. These two structures apparently correspond to the ground states for 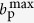 = 4 as shown in the magnified images (cf. Fig. 4g).

With four layers, we observed simple stacking of independent bilayers, just like in the case for small patches (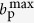 = 3). However, the appearance of the patterns reflects the polymorph of the hexagonal lattice: For the fcc arrangement in Fig. 4e,e’, octahedral hexamers in the upper and lower bilayer partially overlap, resulting in a stripe-like pattern in a two-dimensional image. For hcp in Fig. 4f,f’, on the other hand, the hexamers completely overlap or avoid each other, as seen in the right and left parts of the optical microscopy and simulation image, resulting in a hexagonal or honeycomb-like pattern respectively in a two-dimensional image.

With three layers, the ordered structures are distinct to those described so far: Striped structures appear in Fig. 4c (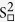) and d (S_Δ_). Their magnified images in Fig. 4c’ show that particles in the second layer form alternate linear clusters with the particles in the first and third layer. For 3□, only the particles in the second layer establish four bonds (see the green patches in the simulation image in Fig. 4c’) and others only three bonds, whereas for 3Δ, the half of the particles in the first and third layer establish four bonds and others only three bonds, as in Fig. 4d’. Thus, inter-patch bonding would be unsaturated, i.e. frustrated, in these ordered states.

As summarized in Fig. 2i for *d* = 1.5 μm and three patch sizes, the states for *θ*_ap_ = 64° correspond to those in Fig. 3 and the states for *θ*_ap_ = 73° and 80° to those in Fig. 4. This result demonstrates that the self-assembled mesostructures by orientational order can be systematically controlled with patch size and confinement thickness.

### Phase behaviour

Most of the experimentally-observed patterns apparently correspond to the ground states with 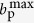 = 3 or 4 when constant ε over a patch is assumed, suggesting that the simple patchy particle model is still applicable to our multilayer system as the monolayer system^16^. However, there are exceptions: The bonding would be unsaturated in the stripe states seen with three layers. In addition, thermal agitation does affect the structure, as shown e.g. by the disturbance in pattern and the appearance of a minor structure in Fig. 2.

**Figure 5:**
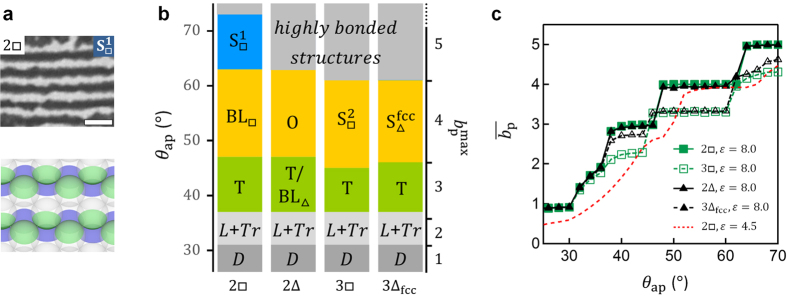
Patch-size and particle-arrangement dependence of orientationally-ordered states in the simulation. (**a**) An optical microscopy image (top) and the simulation image (bottom) of a striped structure in 2□, 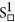 , corresponding to a ground state for 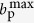 = 5. *d* = 2.0 μm and *θ*_ap_ = 76° in the experiment. The scale bar is 5.0 μm. (**b**) The phase diagram for ε = 8.0 in the simulation, where *θ*_ap_ -step is 2°. The results for four layers and Δ_hcp_ are omitted, because they are almost identical to those for bilayer and Δ_fcc_, respectively. The symbol D denotes dimers, L linear clusters, and Tr triangular trimers (see Supplementary Fig. 3 for the images): These are observed only in the simulation. “/” represents stochastic formation of the states. The highly-bonded structures were not clearly distinguished from each other in the experiment and thus not considered. (**c**) *θ*_ap_ -dependence of the average bond number 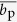 in the simulation for the parameters shown in the graph (see Supplementary Fig. 4 for the other conditions).

Therefore, we studied the phase behaviour in detail using numerical simulation and compared it with the experiment. Firstly, we calculated a phase diagram with a large ε, 8.0, which we expected to produce phases close to the ground state (Fig. 5b). Transitions between the states are clearly indicated by the jumps in the average bond number in a system, 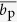, in Fig. 5c. In Fig. 5b, the *θ*_ap_ -ranges of the ordered states almost agree with their geometrical 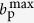-ranges indicated at the right axis (Supplementary Note 3); 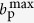 = 3 for the green, 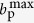 = 4 for the yellow, and 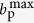 = 5 for the blue regions in the diagram. This agreement shows that the states are close to the ground states which simply maximise 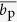, i.e. minimise internal energy, even in 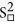 and S_Δ_ states where the bonding is unsaturated. When ε becomes smaller, the transitions in state becomes more continuous and shift to larger *θ*_ap_ due to an entropic effect^9^, as shown in Fig. 5c for a moderate ε, 4.5, in 2□ (cf. Supplementary Fig. 4 for the other conditions).

These simulation results qualitatively explain the patch-size and particle-arrangement dependence in the experiment. Figure 2i corresponds to Fig. 5b for 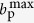 = 3 to 4, suggesting that the ordering in the experiment is predominantly driven by the internal energy, not the rotational entropy, for our experimental conditions. In addition, the correspondence shows that the simple model of patchy particles reproduces the experimental results despite the difference between them described above. The larger *θ*_ap_ in Fig. 2i than in Fig. 5b can be explained by the ε -dependence of the transition as seen in Fig. 5c for 2□, together with the patch thickness dependence of ε in the experiment (Supplementary Note 1). The continuity of the transitions for the smaller ε in Fig. 5c (and Supplementary Fig. 5) explains the patch size dependence of the order in the experiment: The order is lower for *θ*_ap_ = 73° than that for 80° in Fig. 2i. For more quantitative comparison, the modelling in a numerical simulation should reflect more details of the experimental system. The pair potential of interacting particles is not constant even on a patch, and its description requires the van der Waals and Coulombic interaction between dielectric particles with a thin curved finite gold layer with heterogeneity in its thickness, including the effect of ion distributions around them. The particles also possess slight shape anisotropy because of the patch thickness on the spherical surface (see Materials and Methods). Thus, the rotational and translational motion of a particle cannot be completely decoupled in the experiment. Such an effect of shape anisotropy in closely-packed spherical particles has been reported^22^,^23^,^24^. The anisotropy of our particles is, however, much less than that in these reports, playing a considerable role in an almost closest-packed system (see Materials and Methods and Supplementary Fig. 1d); which beyond the scope of this contribution.

## Discussion

The mechanism of orientational ordering is qualitatively explained by the competition between the structure(s) preferred by inter-patch attraction and the specific spatial confinement. Most of the ground states such as T and BL_Δ_ are reported as equilibrium states in a bulk system^12^,^13^, and some of the ground states in bulk are degenerate because they have the same 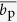, as with the T and BL_Δ_ states for 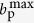 = 3 (Fig. 3b,c), and O and BL_□_ states for 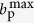 = 4 (Fig. 4a,b): Here it is noteworthy that stacked tetragonal layers correspond to fcc crystal planes perpendicular to the (100) direction, and thus stacked tetragonal and hexagonal layers are indistinguishable in a bulk system. When there are an even number (2 or 4) of layers, the quasi two-dimensionality of the confined system, i.e. quantised number of flat monolayers of particles, and the lack of any patch-substrate interaction select a commensurate state from the degenerate options. For 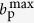 = 3, only the T state is commensurate and thus selected as shown in Figs 3 and 5b, except 2(and 4)Δ. In 2(and 4)Δ, both T and BL_Δ_ states are commensurate and their energetic difference is very small, deriving only from the entropy of spatial arrangement. Thus, they stochastically appear in the simulation with a large ε, and coexist in the experiment with a moderate ε. For 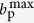 = 4, BL_□_ and O are the only commensurate states in 2(and 4)□ and 2(and 4)Δ, respectively, in agreement with the experimental and simulation results in Figs 2i, 4 and 5.

In contrast, with three layers, none of the ground states in bulk is commensurate for 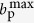 = 4; both O and BL_□_ cannot fill the space. Thus, unique states under confinement, S_Δ_ and 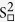, appear (Figs 2i, 4 and 5). In addition, 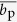 is smaller in three layers than in two layers even for the T state, 37° ≲ *θ*_ap _≲ 40°, in Fig. 5c, indicating that inter-patch bonding is frustrated for 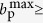
3. The less ordered patterns of the T state in Fig. 3d and e also suggest the incommensurability of the state with three layers. The frustration suggests that orientational ordering with a bilayer periodicity is energetically preferred in closely-packed one-patch particles in a quasi-two-dimensional system.

### Concluding remarks

Our work demonstrates a new and simple means to systematically control self-assembled mesostructures *via* purely orientational ordering in closely-packed spherical patchy particles: The limited spatial dimensionality and boundary conditions by confinement resolve the degeneracy in the ground states or even induce novel structures. The observed ordered structures are predominantly explained by internal energy; however, for smaller inter-patch attraction, there are possibilities that rotational entropy-driven orders appear. There are further possibilities for controlling and obtaining novel orientationally-ordered states by tailoring patch geometry^7^ and using the numerous particle arrangements available from layering transitions^20^,^21^. The essential role of spatial confinement on the orientational ordering revealed here is useful for understanding the ordering behaviour of naturally occurring colloids with anisotropic surface chemistry, such as proteins, confined by interfaces. Finally, closely-packed patchy particles may present a new functional mesosystem through the purely orientational response of its colloidal constituents, where the order would dynamically change against external fields and internal chemical heterogeneity of the solvent.

## Materials and Methods

### Preparation of one-patch particles

Methods for preparing the particles and solvent are almost the same as those in our previous work in ref. 16: A layer of gold was thermally deposited on a hemisphere of monodisperse silica particles (Hyprecica, UEXC) following a 3 nm-thick Chromium layer. Particle diameters are *d* = 0.99, 1.53 and 2.04 μm with dispersion ~3%. The size of a patch, *θ*_ap_, was tuned by chemical etching^25^, and was measured in scanning electron microscopy (SEM) images (Supplementary Fig. 1a,b); its dispersion was ~5%. The patch thickness at the edge is almost zero, and the thickness at its centre was estimated as 30–40 nm^25^. The gold surface was modified with an ionic thiol, sodium-3-mercapto-propanesulfonate (97%, Wako), to prevent irreversible aggregation between the patches. On observation using laser-scanning confocal microscopy, the silica surface of the particle was labelled with a fluorescent dye, Rhodamine B isothiocyanate (283924, Sigma-Aldrich), using the method in ref. 26.

### Sample preparation and observation

The particles were dispersed in a binary mixture of organic solvent (2,6-lutidine, 99%, Sigma-Aldrich) and pure water (18.2 MΩ) at 28.6 ± 0.4 wt% lutidine/water. This solvent induces criticality-induced inter-particle interaction near the critical demixing temperature of the solvent. In this contribution, however, experiments were done at room temperature, which is lower than 10 K from the demixing temperature. Thus, the criticality-induced interaction is negligible^18^,^27^, and the inter-particle interaction is approximated by the DLVO model. We used this system to compare the results with our previous study in a monolayer^16^. The particles dispersed stably for more than a day under these conditions. In the dispersion, a slight amount, typically less than 1%, of flawed particles was observed, such as a particle without a patch, a particle sticking to a substrate, and irreversibly-aggregated particles. Those particles were a cause of structural defects in an ordered state, whereas affecting a state negligibly (e.g. Fig. 2).

The particle dispersion was confined in a wedge-shaped cell composed of two cover glasses with ~0.1% gradient. The glasses were hydrophobised with trimethoxy (octadecyl) silane (90%, Sigma-Aldrich) with the method in ref. 28 to prevent possible patch-substrate attraction. The particles were accumulated on a side of a cell under ~10–20 G centrifugation for more than 30 minutes (Supplementary Fig. 1c). A sample was relaxed for more than a few hours under a weak gravitational pressure in the same direction as the centrifugation by a few % gradient of the cell against horizontal plane. Thus, there is a concentration gradient in a cell because of the pressure gradient after the relaxation: Around the lowest part of the sediment particles they are almost in hard contact with each other and show little thermal motion (e.g. Supplementary Fig. 1d), and around the highest part of the sediment particles dispersed clusters and monomers are in dynamic equilibrium^18^ (e.g. Supplementary Fig. 1e). We observed the region where typical centre-to-centre distance between particles was ≲1.05*d*, i.e. the gap between neighbouring particles was less than 100 nm^16^. The observation was done with an optical microscope (IX73, Olympus) and CFM (TE2000-U, Nikon, with CSU22, Yokogawa).

### Monte Carlo simulation

The simulation methods are almost the same as those in our previous work in ref. 16, where patchy particles in a monolayer were investigated. A standard Monte Carlo simulation was carried out. Particles are arranged into close-packed tetragonal or hexagonal layers. The pairwise attractive potential (depth ε, constant over the area of a patch) works only when two particles are in contact *via* their patches, i.e. both of their patches contain the contact point between the particles. This interaction corresponds to the orientation-dependent part of the Kern-Frenkel model^29^. No interaction with the top and bottom confining surfaces is assumed.

Periodic boundary conditions are adopted in horizontal directions for the rectangular simulation box. 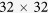 particles are arranged in a tetragonal monolayer. 1152 particles are in a hexagonal monolayer, where an axis of the lattice is parallel to the *y*-axis and 32 particles are arranged in the direction.

In a simulation step, a random rotational move is attempted for a randomly selected particle, and the selection is done *N* times; *N* is the number of particles in a system. The initial orientation of a particle is randomised. For efficient relaxation, the angular range of rotation is changed in a simulation run: For the first quarter of the total steps, the rotation is in ±π, in the second ±π/3, in the third ±π/9, and in the last ±π/30. The number of total simulation steps is ~10^6^–10^8^ dependent on the relaxation rate for a particular parameter set. Four independent simulation runs were carried out for each set of conditions and the results were averaged to give final statistics, as in Fig. 5c.

## Additional Information

**How to cite this article**: Iwashita, Y. and Kimura, Y. Spatial confinement governs orientational order in patchy particles. *Sci. Rep.* **6**, 27599; doi: 10.1038/srep27599 (2016).

## Supplementary Material

Supplementary Information

## Figures and Tables

**Figure 1:**
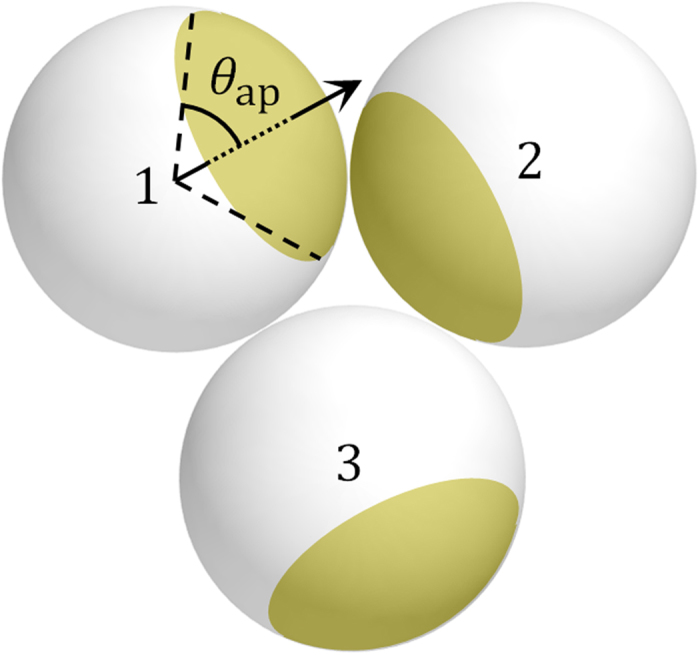
A schematic drawing of closely-packed one-patch particles. A yellow region denotes a patch.
