# Supplementary material for: Spatial confinement governs orientational order in patchy particles
Source: Sci Rep. 2016 Jun 6;6:27599. doi: 10.1038/srep27599 (PMC4893746; doi:10.1038/srep27599)
Supplement: Supplementary Information [file srep27599-s1.pdf]

Supplementary Information for
"Spatial confinement governs orientational order in patchy particles"

Yasutaka Iwashita*, Yasuyuki Kimura

Department of Physics, Kyushu University, 819-0395 Fukuoka, Japan.

*Correspondence to: iwashita@phys.kyushu-u.ac.jp

Contents

Supplementary Figures 1-5

Supplementary Notes 1-3

Supplementary References

Supplementary Figures

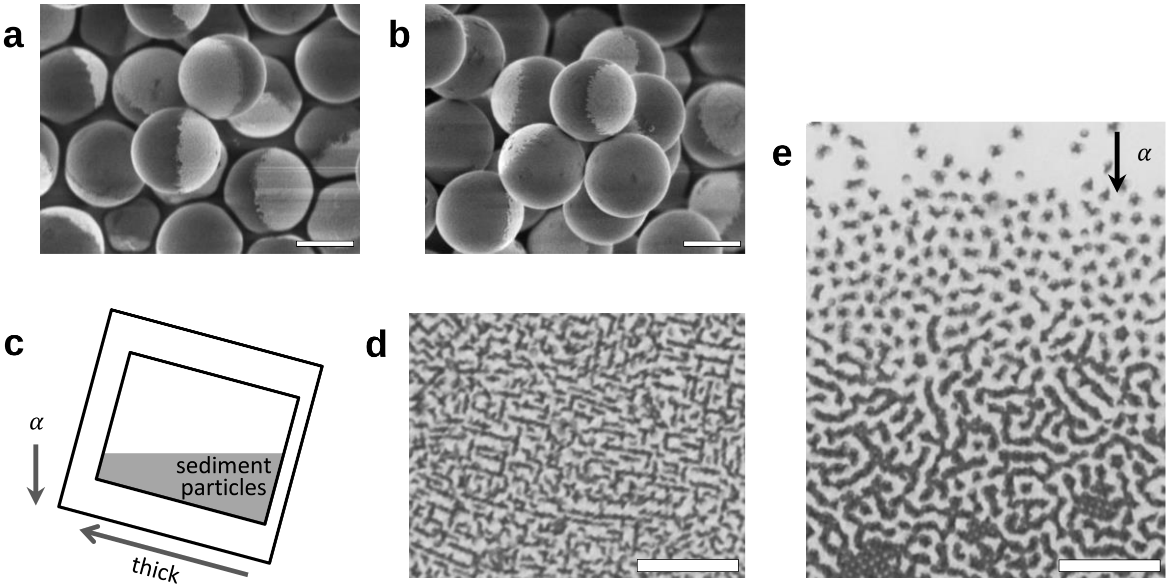

Supplementary Figure 1. Experimental system. (**a** and **b**) SEM images of one-patch colloidal particles for $d = 1.5$ μm. A gold patch appears brighter than the silica surface. Scale bars are 1 μm. θ_{ap} = 80° for (a) and 64° for (b). (**c**) A schematic drawing of a sample cell under centrifugation. The arrow with α indicates the direction of acceleration. (**d** and **e**) Optical microscopy images around the lowest and highest region of the sediment particles, respectively, after relaxation. The confinement thickness is for 2 □ . In (d), because of the closest packing of the particles, small shape asymmetry by the deposited patch hinders their thermal rotational motion. In (e), cluster structures change dependent on the number density of particles. The arrow indicates the direction of acceleration before the relaxation. d = 2.0 μm and θ_{ap} = 76°. Scale bars are 20 μm.

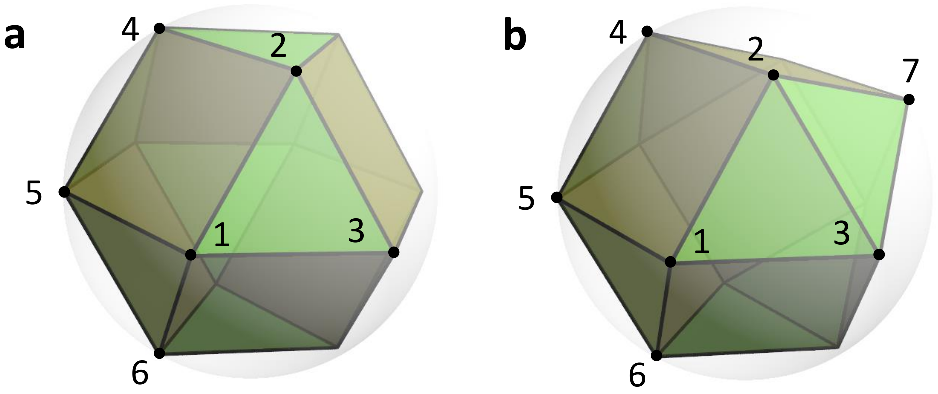

Supplementary Figure 2. Polyhedra whose vertices correspond to the geometry of contact points on a sphere for close packing. (**a**) A cuboctahedron corresponding to the contact points in an fcc crystal. This quasi-regular polyhedron is composed of 8 regular triangles and 6 regular squares, with 24 equal sides and 12 vertices. Six selected vertices are numbered. (**b**) A polyhedron corresponding to the contact points in an hcp crystal, obtained by rotating half of the polyhedron in (a) $\pi/3$ at the hexagonal cross-section containing vertices 2, 3 and 4. Seven selected vertices are numbered.

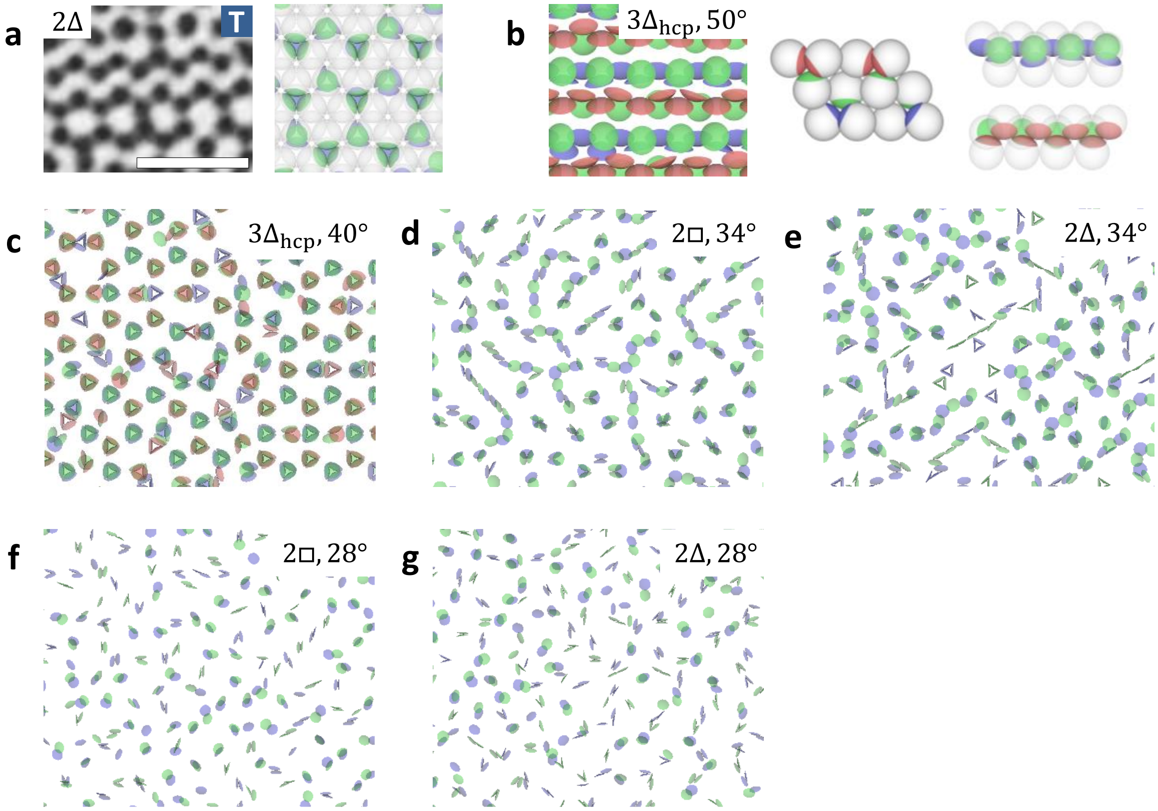

Supplementary Figure 3. Additional images of orientationally-ordered structures. (**a**) Zigzag pattern, appearing in the middle of the optical microscopy image (left) and in the upper part of the simulation image (right). A honeycomb pattern also appears. (**b** to **g**) Images from simulation, where particle arrangement and θ_{ap} are shown on each subfigure. (**b**) Striped pattern, $\mathrm{S}_{\Delta}^{\mathrm{hcp}}$, for $b_{\mathrm{p}}^{\mathrm{max}} = 4$. From the left, top view, side view and separate clusters, respectively. (**c**) Packing of tetrahedral tetramers. (**d** and **e**) Coexistence of linear clusters and triangular trimers, L+Tr in Fig. 5b, for $b_{\mathrm{p}}^{\mathrm{max}} = 2$. (**f** and **g**) Packing of dimers, D in Fig. 5b, for $b_{\mathrm{p}}^{\mathrm{max}} = 1$.

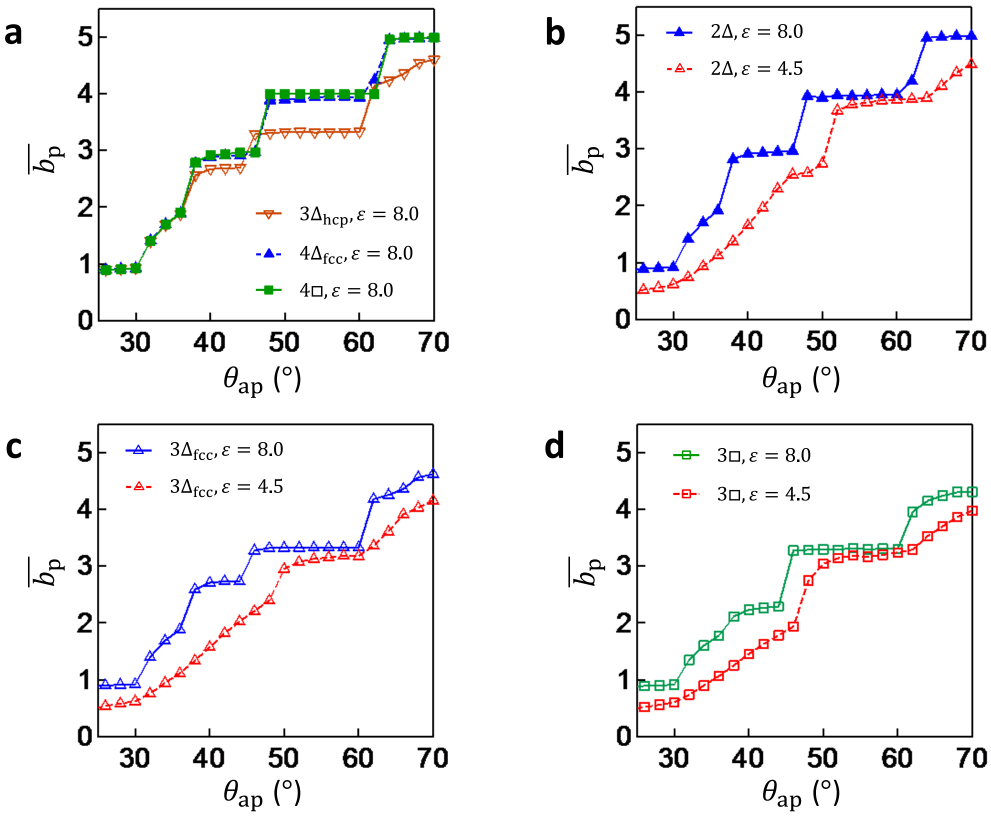

Supplementary Figure 4. Patch-size dependence of average bond number $\overline{b_p}$ for some additional conditions. Their particle arrangements and bonding energies are shown on each graph. In (a), the plot for $4\Delta_{hcp}$ is almost identical to that in $4\Delta_{fcc}$, and is thus omitted. The plots for $\varepsilon = 8.0$ in (b) to (d) are also shown in Fig. 5c.

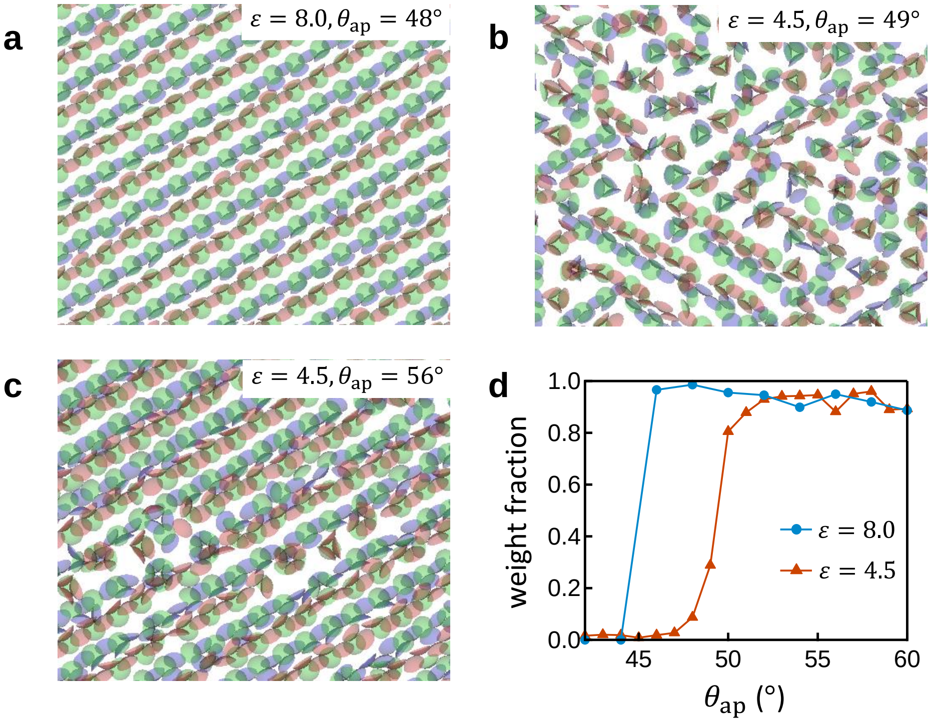

Supplementary Figure 5. Patch-size and inter-patch attraction dependence of S_Δ order in $3\Delta_{fcc}$ in the simulation. (**a** to **c**) Images of the structures. The bonding energy and patch size are shown on the images. (**d**) Plots of the weight fraction of clusters whose size is ≥ 18 particles against patch size for $\varepsilon = 8.0$ and 4.5. The transition from the S_Δ to T state with decreasing θ_{ap} is indicated by the decrease of the large clusters. The transition occurs at larger θ_{ap} for $\varepsilon = 4.5$ than that for $\varepsilon = 8.0$ because of the more significant role of thermal fluctuations in the former.

Supplementary Notes

Supplementary Note 1: Interaction anisotropy in the experiment

In the experiment, as described in the Materials and Methods section, the inter-particle interaction is approximated by the DLVO model. The large van der Waals attraction between gold patches is the origin of the stickiness between patches, or inter-patch attraction ε [1].

Patch thickness continuously decreases from its maximum at the pole to zero at the edge[2]. Thus, van der Waals attraction between patches also decreases to zero when approaching the edge due to the thickness dependence of the attraction[1]. It would make the effective size of a *sticky* patch, i.e. the angular range where the attractive potential is effective compared to thermal energy, smaller than the measured patch size in the experiment. The reason why $S_{\square}^{1}$ was not observed clearly for $d = 1.5\ \mu\text{m}$ in Fig. 2i could be also explained by this non-uniform attraction: ε is almost proportional to *d* from the Derjaguin approximation. Thus, the effective patch size is larger for a bigger particle, and $S_{\square}^{1}$ would have been stabilised only for $d = 2.0\ \mu\text{m}$ in our experimental conditions. In addition, this non-uniformity in ε also suggests that inter-patch bonding energy is not quantised in experiment, which might affect the continuity of the transitions in a phase diagram or the order itself. The thickness of a patch also adds a very small shape anisotropy to the particle, which may also affect the stability of orientationally ordered states slightly.

We emphasise that, nevertheless, the experimentally-observed structures in this contribution are well described with the simple model assuming discretised anisotropy in the inter-particle interaction. More detailed study in the future might reveal the difference due to the modelling.

Supplementary Note 2: Patch size dependence of bond number

Stacked tetragonal layers correspond to fcc crystal planes perpendicular to the (100) direction. Thus, the local particle arrangement in tetragonal and hexagonal layers is that of an fcc or hcp crystal. In an fcc crystal of close-packed spheres, the arrangement of inter-particle contact points on a particle corresponds to the vertices of a cuboctahedron as shown in Supplementary Fig. 2a.

The maximum possible number of bonds per patch, $b_{\mathrm{p}}^{\max}$, is simply determined from the geometry, i.e. how many contact points can be contained within a circular patch. In Supplementary Fig. 2a for an fcc crystal, the minimum patch size for *n* bonds on a patch, $\theta_{\mathrm{ap}}^{\min,n}$, is as follows: $\theta_{\mathrm{ap}}^{\min,2} = 30°$ (a patch covering a side), $\theta_{\mathrm{ap}}^{\min,3} = 35.3°$ (e.g. the 1-2-3 triangle in the figure), $\theta_{\mathrm{ap}}^{\min,4} = 45°$ (e.g. the 1-2-4-5 square), $\theta_{\mathrm{ap}}^{\min,5} = 60°$ (e.g. the two triangles, 1-2-3 and 1-5-6). These minimum patch sizes for $b_{\mathrm{p}}^{\max} = 2$ to 5 are the same in an hcp crystal, because the geometry of the contact point is identical for the $b_{\mathrm{p}}^{\max}$-range as shown in Supplementary Fig. 2b. (In hcp crystals, there are other combinations of vertices for a given bond number. e.g. $b_{\mathrm{p}}^{\max} = 4$

can be attained by the two triangles, 1-2-3-7. However, its minimum patch size, 54.7°, is larger than that of the 1-2-4-5 square. For $n > 5$, $\theta_{ap}^{min,n}$ depends on the crystalline order, fcc or hcp.)

Supplementary Note 3: Stability of a state in the simulation

The commensurability of an ordered structure with periodic boundary conditions could affect its stability, especially when the correlation length of the structure is comparable to or larger than the simulation box size. Although this is the case for many conditions in our simulation, grain boundaries often exist in a calculated structure, proving the stability of the ordered state even when the system geometry is incommensurate with it. Nevertheless, the possible existence of more stable states cannot be completely excluded: In general, numerous metastable states could appear in patchy particles. In particular, an equilibrium state with long spatial periodicity has been found in simulations[3, 4] where stability would strongly depend on the commensurability with the size of the system. In this contribution, the agreement in the selection of ordered structure between simulation and experiment supports the claim that the structures observed in simulation are in equilibrium.

Supplementary References

1. Shemi, O. & Solomon, M. J. Effect of surface chemistry and metallic layer thickness on the clustering of metallodielectric Janus spheres. *Langmuir* **30**, 15408-15415 (2014).
2. Chen, Q. *et al.* Triblock colloids for directed self-assembly. *J. Am. Chem. Soc.* **133**, 7725–7727 (2011).
3. Vissers, T., Preisler, Z. & Smallenburg, F. Predicting crystals of Janus colloids. *J. Chem. Phys.* **138**, 164505 (2013).
4. Preisler, Z., Vissers, T., Munaò, G., Smallenburg, F. & Sciortino, F. Equilibrium phases of one-patch colloids with short-range attractions. *Soft Matter* **10**, 5121-5128 (2014).
